# Role of cinnarizine and nifedipine on anticonvulsant effect of sodium valproate and carbamazepine in maximal electroshock and pentylenetetrazole model of seizures in mice

**DOI:** 10.4103/0976-500X.72348

**Published:** 2010

**Authors:** Ranjana I. Brahmane, Vikrant V. Wanmali, Swanand S. Pathak, Kartik J. Salwe

**Affiliations:** *Department of Pharmacology, MGIMS, Sewagram - 442 102, District Wardha, Maharashtra, India*; 1 *Department of Pharmacology, Mahatma Gandhi Medical College & Research Institute, Pondichery, India*

**Keywords:** Carbamazepine, cinnarizine, nifedipine, seizures, sodium valproate

## Abstract

**Objective:**

To study the effect of calcium channel blockers (CCBs) cinnarizine and nifedipine on maximal electroshock (MES)-induced and pentylenetetrazole (PTZ)-induced convulsions and also their effect in combination with conventional antiepileptic drugs (CAED).

**Materials and Methods:**

For this study, Swiss albino mice were used. Effects of cinnarizine (30 mg/kg), nifedipine (5 mg/kg), sodium valproate (300 mg/kg) and carbamazepine (8 mg/kg) alone and in combination were studied in MES and PTZ seizure models. Abolition of hind limb tonic extension was an index of anticonvulsant activity in MES, while for PTZ seizures, failure to observe even a single episode of tonic spasm for 5 s duration for 1 h was the index. With this, percentage protection was calculated and statistical analysis was carried out using Fisher’s exact test (Ovvind Langsrud software, German version).

**Results:**

In MES seizures, augmented effects were obtained when cinnarizine was combined with sodium valproate, i.e. 100%. In PTZ-induced seizures, augmented effects were obtained when nifedipine was combined with sodium valproate, i.e. 100%. Thus, cinnarizine added to sodium valproate therapy produces significant protection against MES seizures while nifedipine added to sodium valproate therapy produces significant protection against PTZ seizures.

**Conclusion:**

The results provide a lead for potential benefit of adding CCBs to sodium valproate in the treatment of epilepsy, which needs to be explored further.

## INTRODUCTION

Epilepsy is a common and chronic neurological disorder characterized by apparently unprovoked recurrent paroxysmal events or seizures that are associated with a sudden alteration in motor activity and behavior, with or without alteration in conscious awareness. The alteration in state is the result of an abnormal and excessive hypersynchronous firing within a group of epileptic neurons in the brain.[1,2] Epilepsy is the second most common neurological disorder in India.[3,4] Despite many advances in epilepsy research, the pharmacotherapy of epilepsy remains largely empirical, owing to the lack of understanding of the underlying pathology. Moreover, approximately 30% of the people with epilepsy have seizures that do not respond satisfactorily to the conventional antiepileptic drugs (CAEDs).[5] These limitations with the CAEDs alone highlighted the need for exploring the drugs that could potentiate the action of CAEDs so as to make the treatment of epilepsy more effective. Medevite *et al*., has shown the presence of specific binding sites of calcium channel blockers (CCBs) that enable them to cross the blood brain barrier (BBB).[6] This gives an important evidence for the presence of central effects of CCBs.[7] Desmedt *et al*., in 1975, reported that cinnarizine and flunarizine have anticonvulsive properties in rats and mice.[8] The concept of rational polytherapy, which has developed in recent years, is based on the assumption that combining some antiepileptic drugs may results in supraadditive (synergistic) efficacy and infraadditive (antagonistic) toxicity, resulting in an enhanced efficacy/toxicity profile. In order to provide evidence to support this assumption experimentally and because of the above reasons, i.e. CCBs having antiepileptic property, CCBs were combined with established antiepileptic drugs. Flunarizine, a CCB, given along with antiepileptic drugs as add-on therapy has been found to reduce seizure significantly.[9]

Hence, the present study was undertaken to determine whether CCBs like cinnarizine and nifedipine along with antiepileptic drugs could provide superior seizure control in maximal electroshock (MES)-induced and pentylenetetrazole (PTZ)-induced convulsions as compared to CAEDs.

## MATERIALS AND METHODS

### Animals

Male albino mice weighing between 20 and 30 g were used. They were maintained under 12:12 h light:dark cycles and were fed with standard laboratory chow (Ashirwad Industries, laboratory animal diet manufacturer, Punjab, India.) and water *ad libitum*. All the experiments were carried out around the same time each day. Mice not showing convulsions at the time of screening were excluded from the study.

The protocol was duly submitted to and approved by the Institutional Animal Ethical Committee.

### MES seizure method

Anticonvulsant activity was tested for MES seizure by inducing convulsions with an electroconvulsiometer. In this method, electrical stimulation was applied via clipped-ear electrodes (moistened with saline solution before each application) with an electroconvulsiometer, which delivered a constant current of 60 mA for 0.2 s at 60 Hz. Abolition of hind limb tonic extension was taken as an index of anticonvulsant activity.[10] All the drugs were given 45 min prior to the induction of convulsions.

### Chemical method

The chemical convulsant used was PTZ, which was dissolved in normal saline[9] and administered at the dose of 60 mg/kg i.p. All the drugs were given 45 min prior to PTZ. Failure to observe even a single episode of tonic spasm at least for 5 s duration for a period of 1 h was the index of protection.[10]

All the groups received the drugs intraperitoneally at different sites throughout the experiment. Concentration of drugs was so adjusted that all the groups received the same volume of preparation throughout the study. All the drugs were given 45 min prior to the induction of convulsions.

### Effect of cinnarizine and nifedipine against MES-induced seizures

Animals were divided into three groups of 12 animals each. Group 1 was given cinnarizine at the dose of 30 mg/kg while Group 2 received nifedipine at the dose of 5 mg/kg. Group 3 received distilled water and served as the control.

### Effect of cinnarizine and nifedipine against PTZ-induced seizures

Animals were divided into three groups of 12 animals each. Group 1 was given cinnarizine at the dose of 30 mg/kg while Group 2 received nifedipine at the dose of 5 mg/kg. Group 3 received distilled water and served as the control.

### Effect of sodium valproate with CCBs in MES-induced seizures

Animals were divided into three groups of 12 animals each. Group 1 received sodium valproate at the dose of 300 mg/kg and cinnarizine at the dose of 30 mg/kg. Group 2 received sodium valproate at the dose of 300 mg/kg[9] and nifedipine at the dose of 5 mg/kg. Group 3 received sodium valproate at the dose of 300 mg/kg[9] and distilled water and served as control.

### Effect of sodium valproate with CCBs in PTZ-induced seizures

Animals were divided into three groups of 12 animals each. Group 1 received sodium valproate at the dose of 300 mg/kg and cinnarizine at the dose of 30 mg/kg. Group 2 received sodium valproate at the dose of 300 mg/kg[9] and nifedipine at the dose of 5 mg/kg. Group 3 received sodium valproate at the dose of 300 mg/kg[7] and distilled water and served as control.

### Effect of carbamazepine with CCBs in MES-induced seizures

Animals were divided into three groups of 12 animals each. Group 1 received carbamazepine at the dose of 8 mg/kg and cinnarizine at the dose of 30 mg/kg. Group 2 received carbamazepine at the dose of 8 mg/kg and nifedipine at the dose of 5 mg/kg. Group 3 received carbamazepine at the dose of 8 mg/kg and distilled water and served as control.

### Effect of carbamazepine with CCBs in PTZ-induced seizures

Animals were divided into three groups of 12 animals each. Group 1 received carbamazepine at the dose of 8 mg/kg and cinnarizine at the dose of 30 mg/kg. Group 2 received carbamazepine at the dose of 8 mg/kg and nifedipine at the dose of 5 mg/kg. Group 3 received carbamazepine at the dose of 8 mg/kg and distilled water and served as control.

### Statistical analysis

Statistical analysis was carried out using Fisher’s exact test (Ovvind Langsrud software, German version). *P*<0.05 was considered as statistically significant.

## RESULTS

The number of animals protected, number of animals not protected and percentage (%) protection was calculated.

### Effect of cinnarizine and nifedipine against MES- and PTZ-induced seizures

Cinnarizine at a dose of 30 mg/kg provided 50% protection while nifedipine at a dose of 5 mg/kg provided 41.66% protection against MES-induced seizure. Thus, the protection offered was statistically significant in both cinnarizine and nifedipine groups. However, in PTZ-induced seizure, only cinnarizine provided statistically significant protection (33.33%) [Table 1].

**Table 1:** Effect of cinnarizine and nifedipine against MES- and PTZ-induced seizures

Drugs and dose (mg/kg)	MES No. of mice protected n=12 (%protection)	PTZ No. of mice protected n=12 (%protection)
Control (distilled water)	0 (00.00)	0 (00.00)
Cinnarizine (30 mg/kg)	6 (50.00)*	4 (33.33)*
Nifedipine (5 mg/kg)	5 (41.66)*	2 (16.66)

* *P*<0.05 when compared to control

### Effect of sodium valproate with CCBs in MES- and PTZ-induced seizures

Sodium valproate and cinnarizine offered 100.0% protection while sodium valproate and nifedipine offered 83.33% protection against MES-induced seizure, which was statistically significant compared to protection offered by sodium valproate alone (50%). Similarly, sodium valproate and cinnarizine offered 83.33% protection while sodium valproate and nifedipine offered 100.00% protection against PTZ-induced seizure, which was again statistically significant as compared to only 50.0% protection offered by sodium valproate alone [Table 2].

**Table 2:** Effect of sodium valproate with CCBs in MES- and PTZ-induced seizures

Drugs and dose (mg/kg)	MES No. of mice protected n=12 (% protection)	PTZ No. of mice protected n=12 (% protection)
Sodium valproate (300 mg/kg)	6 (50.00)	6 (50.00)
Sodium valproate (300 mg/kg) and cinnarizine (30 mg/kg)	12 (100.00)*	10 (83.33)*
Sodium valproate (300 mg/kg) and nifedipine (5 mg/kg)	10 (83.33)*	12 (100.00)*

* *P*<0.05 when compared to sodium valproate

### Effect of carbamazepine with CCBs in MES- and PTZ-induced seizures

Carbamazepine and cinnarizine or carbamazepine and nifedipine did not offer any significant protection either against MES-induced or against PTZ-induced seizure as compared to protection offered by carbamazepine alone [Table 3].

**Table 3:** Effect of carbamazepine with CCBs in MES- and PTZ-induced seizures

Drugs and dose (mg/kg)	MES No. of mice protected n=12 (% protection)	PTZ No. of mice protected n=12 (% protection)
Carbamazepine (8 mg/kg)	6 (50.00)	7 (58.33)
Carbamazepine (8 mg/kg)and cinnarizine (30 mg/kg)	6 (50.00)	8 (66.66)
Carbamazepine (8 mg/kg)And nifedipine (5 mg/kg)	6 (50.00)	8 (66.66)

## DISCUSSION

An important characteristic of all CCBs is their ability to inhibit the inward flow of calcium ions. CCBs depress the epileptic depolarization of neurons. In this study, it was observed that cinnarizine and nifedipine have an anticonvulsant action. In MES seizures, the percent protection of cinnarizine is 50% and of nifedipine is 41.66%. All these drugs prolong the latent period and reduce the duration of tonic extensor phase of MES.[11] In PTZ-induced seizures, the percent protection of cinnarizine is 33.33% and of nifedipine is 16.66%. They also reduce the occurrence of convulsions and the number of deaths due to PTZ. In this study, it was observed that CCBs have an anticonvulsant action.

According to Tartara,[12] the possible mechanism of the anticonvulsant action of CCBs is the potent antagonism of transmembrane calcium influx in cerebral neurons and disturbances in neuronal calcium conductance, which is implicated in the generation and propagation of seizure activity. In combined drug therapy in MES, augmented effects were obtained when cinnarizine and nifedipine were added to CAEDs and 100% seizure control was obtained when cinnarizine was combined with sodium valproate. Hence, combination of cinnarizine with sodium valproate can be effective in grand mal epilepsy.

In PTZ-induced seizures, augmented effects were obtained when cinnarizine and nifedipine were combined with carbamazepine and 100% protection was obtained when nifedipine was combined with sodium valproate. Hence, the combination of nifedipine with sodium valproate can be effective in Petit Mal seizures.

In case of sodium valproate, two general hypotheses have been proposed, viz. blockade of voltage-dependent sodium channels and enhancing the gamma-aminobutyric acid (GABA) -mediated inhibition. Similarly, CCBs like cinnarizine have been claimed to have an effect on voltage-sensitive sodium channels.[13] This is due to the cerebrovascular effect of cinnarizine, which could provide a direct neuroprotective effect against the damaging influx of calcium and also prevent neuronal damage as a result of MES- and PTZ-induced seizures.[14] There is a possibility that cinnarizine also acts by interacting with voltage-dependent sodium channels.[15] Mcdevitt *et al*.[6] suggested that calcium antagonists like nifedipine and others are lipid soluble and can therefore cross the BBB with relative ease. Considerable evidences have been gathered that nifedipine blocks glutamate receptors found in the central nervous system that is responsible for neuronal injury observed after ischemia, trauma, epilepsy and several types of neurodegenerative maladies.[16] However, further studies are required to establish the exact mechanism of action of CCBs in epilepsy and clinical studies to establish their use in human population.

## CONCLUSION

Thus, the concept of rational polytherapy in treatment of epilepsy needs consideration. The results obtained provide a lead for the possible potential benefit of adding CCBs to sodium valproate in the treatment of epilepsy, which needs to be explored further.
